# Development of a consortium-based microbial agent beneficial to composting of distilled grain waste for *Pleurotus ostreatus* cultivation

**DOI:** 10.1186/s13068-021-02089-4

**Published:** 2021-12-17

**Authors:** Sibao Wu, Rongrong Zhou, Yuting Ma, Yong Fang, Guopai Xie, Xuezhi Gao, Yazhong Xiao, Juanjuan Liu, Zemin Fang

**Affiliations:** 1 School of Life Sciences, Anhui University, Hefei, 230601 Anhui China; 2 Anhui Key Laboratory of Modern Biomanufacturing, Hefei, 230601 Anhui China; 3 Anhui Golden Seed Winery Co., LTD, Fuyang, 341200 Anhui China; 4 Livestock and Poultry Breeding Service Center of Fuyang City, Fuyang, 341200 Anhui China

**Keywords:** *Pleurotus ostreatus* cultivation, Microbial agent, Distilled grain waste, Composting, Microbial metabolism

## Abstract

**Background:**

*Pleurotus ostreatus* is an edible mushroom popularly cultivated worldwide. Distilled grain waste (DGW) is a potential substrate for *P. ostreatus* cultivation. However, components in DGW restrict *P. ostreatus* mycelial growth. Therefore, a cost-effective approach to facilitate rapid *P. ostreatus* colonization on DGW substrate will benefit *P. ostreatus* cultivation and DGW recycling.

**Results:**

Five dominant indigenous bacteria, *Sphingobacterium* sp. X1, *Ureibacillus* sp. X2, *Pseudoxanthomonas* sp. X3, *Geobacillus* sp. X4, and *Aeribacillus* sp. X5, were isolated from DGW and selected to develop a consortium-based microbial agent to compost DGW for *P. ostreatus* cultivation. Microbial agent inoculation led to faster carbohydrate metabolism, a higher temperature (73.2 vs. 71.2 °C), a longer thermophilic phase (5 vs. 3 days), and significant dynamic changes in microbial community composition and diversity in composts than those of the controls. Metagenomic analysis showed the enhanced microbial metabolisms, such as xenobiotic biodegradation and metabolism and terpenoid and polyketide metabolism, during the mesophilic phase after microbial agent inoculation, which may facilitate the fungal colonization on the substrate. In accordance with the bioinformatic analysis, a faster colonization of *P. ostreatus* was observed in the composts with microbial inoculation than in control after composting for 48 h, as indicated from substantially higher fungal ergosterol content, faster lignocellulose degradation, and higher lignocellulase activities in the former than in the latter. The final mushroom yield shared no significant difference between composts with microbial inoculation and control, with 0.67 ± 0.05 and 0.60 ± 0.04 kg fresh mushroom/kg DGW, respectively (*p* > 0.05).

**Conclusion:**

The consortium-based microbial agent comprised indigenous microorganisms showing application potential in composting DGW for providing substrate for *P. ostreatus* cultivation and will provide an alternative to facilitate DGW recycling.

**Supplementary Information:**

The online version contains supplementary material available at 10.1186/s13068-021-02089-4.

## Background

*Pleurotus ostreatus*, commonly known as the oyster mushroom, is cultivated worldwide and has become the second most popularly cultivated edible mushroom during the last 10 years [1]. Many reasons are responsible for the production increase. Among them, the economic, ecological, and medicinal benefits are highlighted [2]. Remarkably, *P. ostreatus* requires a shorter growth time when compared to other edible mushrooms and grows on a broad range of natural substrates from woodland, agricultural, and animal husbandry [1, 3]. Various agricultural and agro-industrial by-products, such as straw, grass, sawdust, coffee pulp, and corncob are suitable substrates for *P. ostreatus* cultivation. Usually, materials from local places are recommended for cultivators from different countries to lower the costs of cultivation substrates. For example, coffee husk was practiced in Brazil to cultivate oyster mushrooms [4]. In Asian countries, sorghum, coffee pulp, cottonseed hulls, and wheat straw are popular materials used in *P. ostreatus* cultivation [1].

Distilled grain waste (DGW), the primary by-product of the Chinese spirit-making process, is produced and discharged as the primary solid waste [5, 6]. Approximately 25 million tons of DGW have been generated annually in China in the past 5 years. The traditional disposal of DGW into landfills, incineration to produce steam, or directly be utilized as fertilizer caused resource wastes and has resulted in severe environmental problems, including water and soil pollution, land devaluation, and undesirable odor production [7], because the DGW has high water content (55‒60%) and organic matter content (80% of the dry weight), and easy to decay [8]. DGW is rich in carbohydrates, proteins, amino acids, vitamins, and microelements from microorganisms and fermented residues of sorghum, corn, wheat, rice, and rice husk [9]. Therefore, it is a suitable raw substrate for *P. ostreatus* cultivation. Thus, the proper and sustainable treatment of DGW to cultivate *P. ostreatus* will be one of the best solutions to facilitate the DGW re-utilization.

Fermentation material based on the composting of raw materials is widely practiced worldwide to facilitate mushroom cultivation due to the advantages of low pollution, low cost, and simple process [2, 10]. Microorganisms are one of the critical factors that affect composting. To promote the composting process, some researchers inoculate functional strains in the substrates to accelerate the composting process and increase the nutrient content of the substrates. For example, the degree of aromaticity and stability of dissolved organic matter and humic substances were substantially enhanced after inoculation of a multifunctional thermophilic microbial consortium in manure–sugarcane leaf composting [11]. Addition of bacteria to cattle manure compost promoted microbial activity and the degradation of cellulose-rich waste [12]. However, in most studies, commercial general agents or strains from microbial collections were inoculated into compost. It is difficult to ensure whether the foreign strains could adapt to the local environment of a specific compost. As a result, several studies have screened functional strains from natural compost to ensure that the microorganisms quickly adapt to the environment [13, 14]. Although studies were taken on optimizing DGW composting conditions for biofertilizer preparation [8], no investigation was carried out to evaluate the application potential of microbial agents on composting DGW for *P. ostreatus* cultivation.

In the present study, indigenous bacterial strains were isolated from DGW samples, based on which a consortium-based microbial agent containing *Sphingobacterium* sp. X1, *Ureibacillus* sp. X2, *Pseudoxanthomonas* sp. X3, *Geobacillus* sp. X4, and *Aeribacillus* sp. X5 was developed to compost DGW for *P. ostreatus* cultivation. Given that the temperature determines microbial activities and is correlated with composting efficiency [15], DGW samples from the thermophilic phase of composting were analyzed and used to isolate beneficial bacteria. The effects of the microbial agent inoculation on DGW composting and *P. ostreatus* colonization and growth were evaluated. The high-throughput sequencing technique was also used in this research to evaluate microbial community succession and metabolism after microbial agent inoculation.

## Results and discussion

### Screening bacteria from distilled grain waste compost for microbial inoculation

**Fig. 1:**
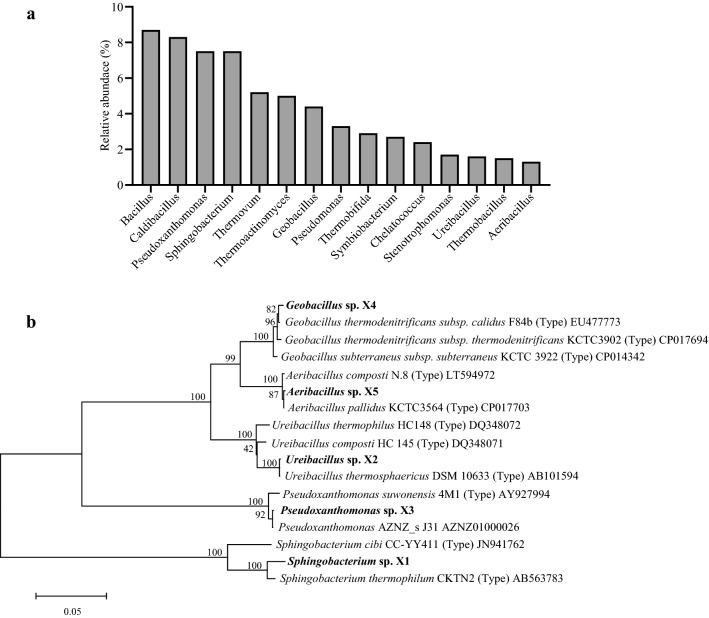
The identification of microorganisms in DGW composting. **a** The top 15 genera of microorganisms in DGW composting of the thermophilic phase. **b** The phylogenetic tree of the five screened microorganisms was constructed using the MEGA 7 program based on the maximum likelihood method

In order to obtain indigenous microorganisms to develop a microbial agent for DGW composting, samples from the thermophilic phase of DGW composting were used as the source of beneficial bacteria [15]. Simultaneously, high-throughput sequencing was employed to analyze the dominant microorganisms in DGW compost to guide the strain isolation. Results revealed that *Thermobacillus*, *Thermoactinomyces*, *Stenotrophomonas*, *Pseudomonas*, *Symbiobacterium*, *Pseudoxanthomonas*, *Ureibacillus*, *Caldibacillus*, *Sphingobacterium*, *Thermobifida*, *Aeribacillus*, *Bacillus*, *Geobacillus*, *Chelatococcus*, and *Thermovum* were the top 15 genera in the thermophilic phase of DGW compost (Fig. 1a). The bacterial genera can be more or less abundant depending on starting material, composting procedure, and analytical methodologies. For example, the dominant bacterial genera, including *Pseudomonas*, *Sphingomonas*, *Bacillus*, *Geobacillus*, *Ureibacillus*, *Pseudoxanthomonas*, and *Thermobispora*, were identified from composted *P. ostreatus* substrate when using wheat straw–alfalfa mixture as the starting material [16], while *Acinetobacter*, *Pseudoxanthomonas*, *Sphingobacterium*, *Terribacillus*, *Thermobacillus*, and *Thermobispora* were the dominant microbial genera in sugarcane straw compost [17].

DGW samples were diluted to 10^–8^ and spread on LB plates to ensure that the dominant microorganisms in compost samples were efficiently isolated. Finally, five strains with different colony phenotypes were selected and utilized in the following experiments. The strains identified were *Sphingobacterium* sp. X1, *Ureibacillus* sp. X2, *Pseudoxanthomonas* sp. X3, *Geobacillus* sp. X4, and *Aeribacillus* sp. X5 based on 16S rRNA gene sequence similarities (16S rDNA GenBank No. MZ323963-MZ323967) and belonged to the top fifteen genera as mentioned above (Fig. 1b, Additional file 1: Table S1). These five species are well known to utilize complex organic matter, such as polysaccharides, phenolic compounds, and polycyclic aromatic hydrocarbons [18–22]. For example, *Sphingobacterium* sp. is a promising candidate for lignocellulolytic enzymes [22] and acenaphthene utilization [23]. *Ureibacillus* sp. is well known for its high efficiency in removing phenolic compounds, its production of a broad range of lignocellulolytic enzymes, including laccases, tyrosinase, peroxidases, catalases, and oxidases, and efficiently degrading furfural and 5-hydroxymethylfurfural with minor sugar consumption (< 5%) [24]. *Pseudoxanthomonas* sp. is the key diazotrophic community influencing NH_4_^+^-N transformation in dairy manure and corn stalk compost [25]. *Geobacillus* sp. can degrade and metabolize hemicellulose [26]. Based on the dual culture experiments on LB agar plates, these five strains showed no or trace competition between each other (data not shown). Thus, these five newly isolated strains were then employed to develop a consortium-based agent.

### Complex microbial agent inoculation improves physicochemical properties of DGW compost

**Fig. 2:**
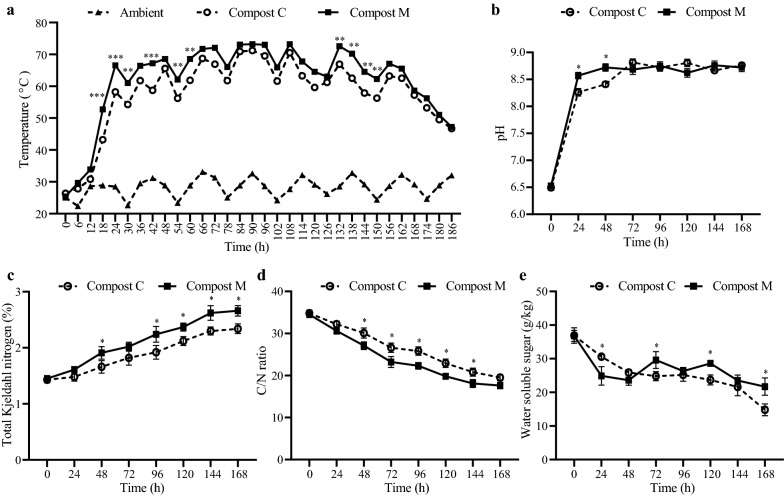
The physicochemical properties of DGW composting with and without microbial inoculation. **a** Temperature. **b** pH. **c** Total Kjeldahl nitrogen. **d** C/N ratio. **e** Water soluble sugar content. *p < 0.05, **p < 0.01, ***p < 0.001. Compost M, with microbial inoculation. Compost C, without microbial inoculation

The physicochemical properties of Compost M (with microbial inoculation) and Compost C (control without microbial inoculation) were analyzed to evaluate the effect of the microbial agent inoculation on composting and summarized in Fig. 2. The internal temperature of the compost pile rapidly increased to over 65 °C within 24 h in Compost M, one day earlier than that in Compost C (Fig. 2a). Although the temperature curves and the duration of the thermophilic phase (> 50 °C) between the two groups were similar, the temperature of Compost M reached and kept at the peak of 73.2 °C, in comparison to that of 71.2 °C in Compost C. Temperature can be used to evaluate the quality of composting [15] and reflects microbial activity during the composting process, especially at the early phase [27]. Thus, our results indicated that the microorganisms in Compost M were more active than those in Compost C. The acceleration of composting temperature might improve compost quality because this biological self-heat generation process is essential for killing pathogens and hastening maturity during composting [28]. Other studies also observed increased temperatures after adding extra microorganisms. For example, Chi et al. [29] revealed that inoculating swine manure and rice straw co-compost with *Streptomyces griseorubens* JSD-1 contributed to high temperatures (maximum 66.8 °C). Meanwhile, the high temperature caused moisture evaporation, leading to a porosity increase in composting feedstock and maintaining sufficient oxygen in the heap. Both the increase in temperature and oxygen content promoted the activity of microorganisms and accelerated the degradation of organic substances [30].

The pH value of Compost M rose faster than that of Compost C from about 6.5 to 8.5 at the initial stage of the composting process. Subsequently, pH of both groups remained at 8.5–8.9 without significant difference (*p* > 0.05, Fig. 2b). The increase in pH was attributed to volatile ammonia and ammonium produced by organic nitrogen decomposition [31]. Total Kjeldahl nitrogen (TKN) concentration increased in both composts because of the weight reduction of compost mass caused by organic degradation (Fig. 2c). However, the C/N ratio was reduced in the two groups, indicating that carbon decomposition was faster than nitrogen loss (Fig. 2d). Simultaneously, the higher TKN concentration but less C/N ratio in Compost M than Compost C also suggested that organic matter was consumed faster in Compost M, indicating a more robust microbial metabolism in Compost M [32]. Following this, the content of water soluble sugar (WSS) rapidly decreased in Compost M between 0 and 48 h (Fig. 2e). On the other hand, WSS content increased from 48 to 72 h in Compost M. This phenomenon may be due to the fast hydrolyzation of carbohydrates such as cellulose and hemicellulose [33]. In the end, WSS content in Compost M was higher than that in Compost C at 168 h (*p* < 0.05, Fig. 2e).

In total, our results showed that inoculation with a complex microbial agent caused changes in physicochemical properties of DGW compost which is a common phenomenon observed during composting of substrates with other microbial agents. For example, after inoculating a thermophilic bacterium (*Geobacillus stearothermophilus* CHB1) in a sludge compost, Fang and the coworkers found that the high-temperature stage (> 50 °C) of the CHB1 inoculated compost and control compost without inoculum started on day 5 and 8, respectively. Furthermore, at the end of composting, the CHB1 inoculated compost showed a higher loss of total organic carbon, lower C/N ratio, and lower moisture content [34].

### Complex microbial agent changed bacterial community diversity and composition

The microbial ecology of the substrate is the underlying driver of the composting process. However, the bacterial community composition of oyster mushroom substrate preparation via composting is still poorly understood [17]. Here, the bacterial community diversity of the two composting groups was analyzed at mesophilic, thermophilic, and cooling phases to obtain deep insights into variations in microbial composition and illustrate the effects of the microbial agent inoculation on composting. After the preliminary quality filtering, an average read depth of 76,323 reads per sample was obtained and clustered to amplicon sequence variants (ASV) at a high similarity level (≥ 97%). PCoA analysis based on ASV abundance was performed to gain an overview of microbial composition. The microorganisms from the different composting phases were distanced from each other (Additional file 1: Fig. S1a), indicating that temperature played an essential role in the succession of microbial communities [35]. Bacterial community richness slightly decreased during the thermophilic phase in Compost M comparing to Compost C as indicated by Chao 1 index (Additional file 1: Fig. S1b). By comparison, in Compost M, the bacterial community diversity represented by the Shannon index slightly increased (Additional file 1: Fig. S1c). These results suggested that the inoculation of microorganisms altered the *α*-diversity of the bacterial community during the composting process, which may explain the rapid increase in temperature and the extended thermophilic phase in Compost M because microbial diversity correlates with the strong mineralization of organic matter [36, 37].

**Fig. 3:**
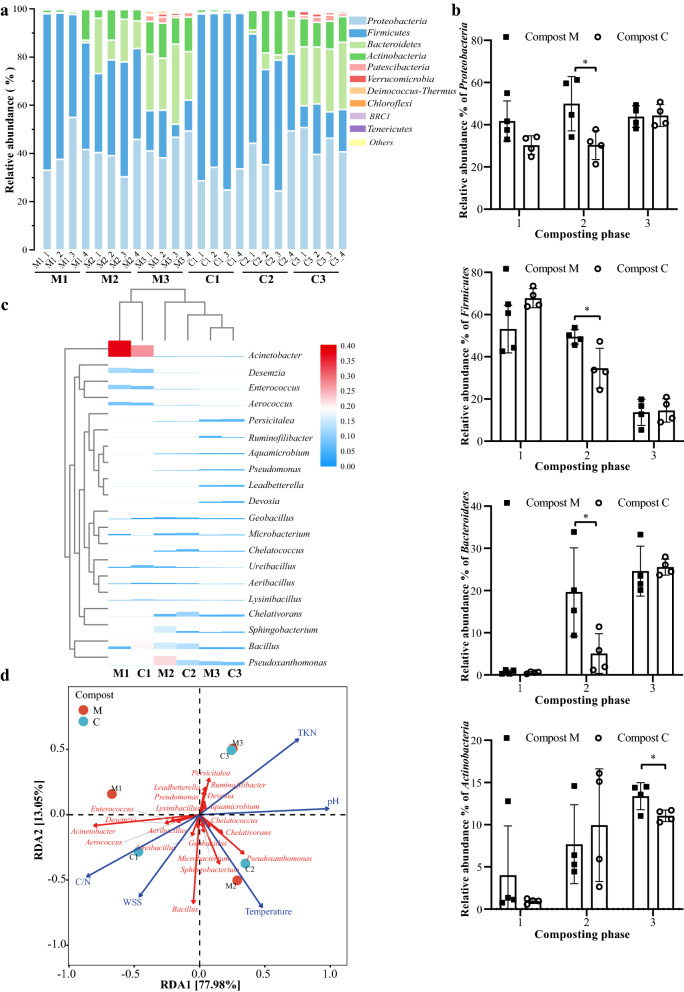
Changes in bacterial community composition during composting in DGW samples with and without microbial inoculation. **a** Phylum level. **b** The relative abundance of *Proteobacteria*, *Firmicutes*, *Bacteroidetes*, and *Actinobacteria*. **c** The heatmap of top 20 genera in DGW samples. **d** RDA of compost physiochemical properties with bacterial communities. M1, M2, and M3 represent the samples from mesophilic, thermophilic, and cooling phases of Compost M, respectively, while C1, C2, and C3 represent the samples from mesophilic, thermophilic, and cooling phases of Compost C. *p < 0.05

Four phyla, namely *Firmicutes*, *Proteobacteria*, *Bacteroidetes*, and *Actinobacteria*, are most dominant in composting processes [38], and comprised the dominant taxa in all samples, accounting for 95.48–99.65% at the different composting phases (Fig. 3a). In Compost C, the relative abundance of *Proteobacteria* was negatively correlated with temperature, similar to a microbial inoculated compost with pig manure as the start material [36]. The abundance increased from about 30.3% during the mesophilic and thermophilic phases to 44.3% during the cooling phase (Fig. 3b). By contrast, this proportion did not considerably change at the three phases in Compost M. Consistent with some previous studies [39, 40], the composting process decreased the proportion of *Firmicutes* but increased that of *Bacteroidetes* and *Actinobacteria* in both groups (Fig. 3b–d). Given that the inoculated bacteria were from 72 h of the thermophilic phase, the relative abundances of *Proteobacteria* (19.6%, *p* < 0.05), *Firmicutes* (14.9%, *p* < 0.05), and *Bacteroidetes* (14.6%, *p* < 0.05) were significantly higher in Compost M than those in Compost C during the thermophilic phase. *Firmicutes*, a classic fermenting group of bacteria [41], form heat-resistant endospores during the thermophilic phase [42]. Other members, such as *Bacteroidetes*, are responsible for breaking down lignocellulosic plant polysaccharides including hemicellulose and cellulose, and subsequently releasing short-chain fatty acids [43]. Thus, we speculate that the increase in the relative abundance of these three phyla led to a higher composting temperature and a faster carbohydrates consumption. Moreover, *Actinobacteria* was relatively abundant in Compost M during the cooling stage (*p* < 0.05).

At the genus level, *Acinetobacter*, *Desemzia*, *Enterococcus*, and *Aerococcus* were abundant in both groups during the mesophilic phase, accounting for 67.98 and 51.00% of the total genera in Compost M and C, respectively (Fig. 3c). They gradually died during the thermophilic phase because of their sensitivity to heat [44]. *Bacillus*, *Chelativorans*, especially *Sphingobacterium* (15.57%) and *Pseudoxanthomonas* (21.67%), which were very few at the mesophilic phase, became the dominant genera during the thermophilic phase because of their heat resistance. These genera are decomposers and participate in carbon and nitrogen cycles [45, 46], and most of them were more abundant in Compost M than in Compost C. For example, *Sphingobacterium*, belongs to *Bacteroidetes* and is closely related to carbon and nitrogen cycles [47]. When the composting process entered the cooling stage, some exogenous microorganisms, including *Pseudomonas*, *Ruminofilibacter*, *Devosia*, *Persicitalea*, and *Leadbetterella* entered and proliferated in the samples. *Pseudomonas* is widely distributed in nature and can decompose complex polymers, such as lignocellulose [48], *Ruminofilibacter* can degrade xylan [49], and *Devosia* is a genus of cellulolytic bacteria [50]. These genera may contribute to lignocellulose degradation during DGW composting.

Redundancy analysis (RDA) was conducted to explore correlations between bacterial communities and physicochemical properties, including temperature, pH, TKN, C/N, and WSS (Fig. 3d). The bacteria during the three composting phases were divided into three clusters. The correlations were ranked in the following order: C/N (r^2^ = 0.9876, *p* = 0.0083) > temperature (r^2^ = 0.9434, *p* = 0.0347) > pH (r^2^ = 0.9217, *p* = 0.0236) > TKN (r^2^ = 0.9099, *p* = 0.0375) > WSS (r^2^ = 0.9064, *p* = 0.1). Specifically, the C/N ratio was positively associated with *Acinetobacter*, *Desemzia*, *Enterococcus*, and *Aerococcus*. These genera were relatively abundant during the mesophilic phase, responsible for substrate degradation [17], and then died (Fig. 3c). Temperature showed positive relationships with *Aeribacillus*, *Ureibacillus*, *Geobacillus*, *Bacillus*, *Microbacterium*, *Sphingobacterium*, *Pseudoxanthomonas*, *Chelatococcus*, and *Chelativorans*, which are thermophilic bacteria and can produce different extracellular decomposing enzymes for degradation of carbohydrates and cellulose [45, 46, 51–53].

### Complex microbial agent changed the potential function of bacterial community

**Fig. 4:**
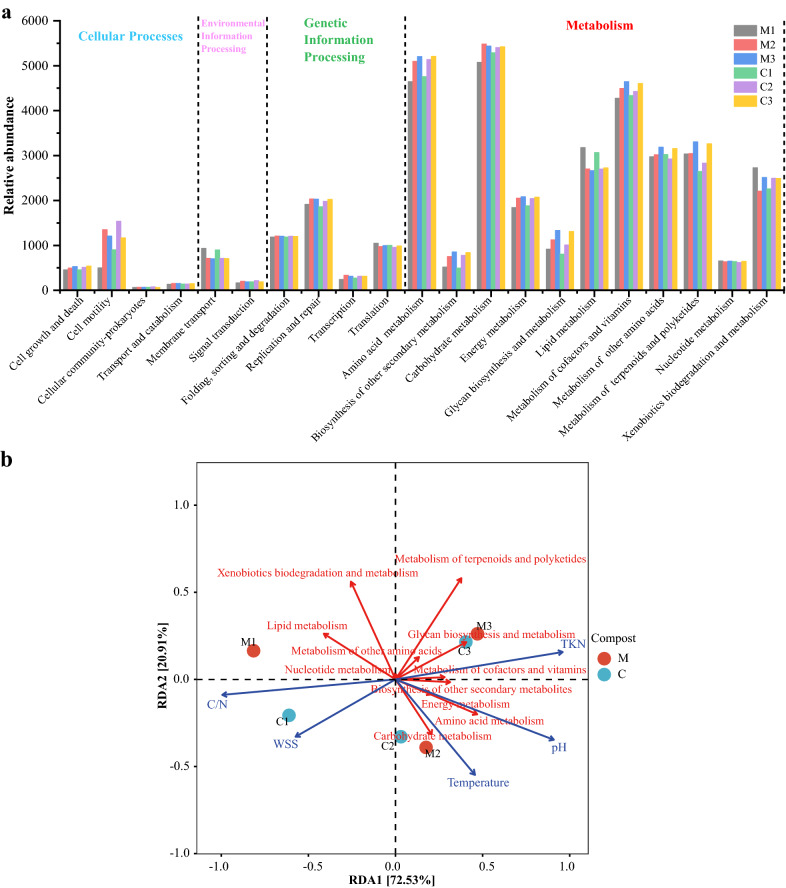
Comparison of the potential functions of bacterial community during composting between DGW samples. **a** The level 2 KEGG microbial ortholog function profiles at different composting stages. **b** RDA of compost physiochemical properties with bacterial communities. M1, M2, and M3 represent the samples from mesophilic, thermophilic, and cooling phases of Compost M, respectively, while C1, C2, and C3 represent the samples from mesophilic, thermophilic, and cooling phases of Compost C

Four main bacterial functions, including metabolism (eleven pathways), genetic information processing (four pathways), environmental information processing (two pathways), and cellular processes (four pathways), were enriched in the samples from both groups (Additional file 1: Fig. S2). Most predicted functional genes during the DGW composting process were assigned to metabolism (80.22–81.75% in Compost M, 79.86–81.19% in Compost C), which is associated with organic matter degradation [54, 55]. Distilled grain waste is rich in starch and lignocellulose. Similar to previous studies on lignocellulosic composting [54], the genes related to the metabolism of carbohydrates, amino acids, cofactors, and vitamins accounted for the top three highest proportions during DGW composting. Their relative abundance substantially increased from the mesophilic phase to the thermophilic phase, probably because of the degradation of easily degradable compounds, such as starch and protein [56]. Carbohydrate metabolism was higher in Compost M than in Compost C during the thermophilic phase. This phenomenon may attribute to the increase in some microbes caused by the inoculants (Fig. 4a). Furthermore, the increase in decomposers affected various complex compounds, such as cellulose and hemicellulose; thus, the metabolism of carbohydrates, amino acids, cofactors, and vitamins remained high during the cooling phase. Unlike that in Compost C, xenobiotic biodegradation and metabolism, as well as terpenoid and polyketide metabolism, accelerated in Compost M during the mesophilic phase (Fig. 4a). The lipid metabolism was active in both groups because of the high fatty acid content of DGW. The accelerated xenobiotic biodegradation and metabolism and lipid metabolism during the mesophilic phase of Compost M contributed to the degradation of complex components, such as alcohols, phenols, and aldehydes, in DGW, producing small molecular nutrition for *P. ostreatus* mycelial growth [2].

RDA correlating the metabolic functions and physicochemical properties further confirmed this fact (Fig. 4b). The correlations were ranked in the following order: C/N (r^2^ = 0.9953, *p* = 0.0013) > temperature (r^2^ = 0.9461, *p* = 0.0111) > pH (r^2^ = 0.9030, *p* = 0.05) > WSS (r^2^ = 0.8292, *p* = 0.0708) > TKN (r^2^ = 0.5385, *p* = 0.3055). The C/N ratio was positively associated with lipid metabolism and xenobiotic biodegradation and metabolism. Temperature was associated with the metabolism of amino acids, carbohydrates, and energy. Therefore, regulating the C/N ratio in composting can effectively promote the metabolism of microbial communities, increase temperature, and promote the degradation of organic matter (Fig. 3d and 4b).

**Fig. 5:**
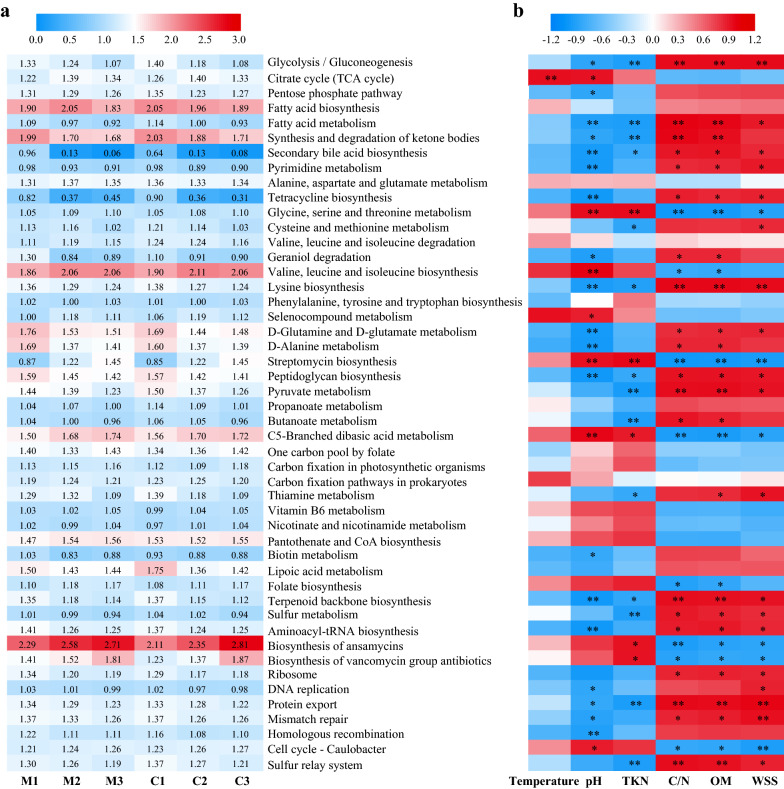
KEGG categories differed significantly between DGW samples and their correlation with physicochemical properties. **a** The level 3 KEGG ortholog function predictions of the relative abundance of the top 50 metabolic pathways. **b** Pearson correlation analysis between metabolic pathway in **a** and physicochemical properties of compost. M1, M2, and M3 represent the samples from mesophilic, thermophilic, and cooling phases of Compost M, respectively, while C1, C2, and C3 represent the samples from mesophilic, thermophilic, and cooling phases of Compost C. *p < 0.05, **p < 0.01

The top 50 enriched KEGG function terms are shown in Fig. 5a. Among them, the biosynthesis of ansamycin, synthesis and degradation of ketone bodies, fatty acid biosynthesis, and valine, leucine, and isoleucine biosynthesis accounted for the most active pathways at all periods of DGW composting (Fig. 5a). The abundance of genes related to ansamycin synthesis in Compost M was enriched in the first two phases compared with Compost C. Ansamycin production can help defeat mycoviruses commonly infected by *P. ostreatus* and promote its fruit body formation [57]. Moreover, genes related to glycolysis/gluconeogenesis, citrate cycle (TCA cycle), pentose phosphate pathway, pyruvate metabolism, and C5-branched dibasic acid metabolism were also accounted for a relatively higher proportion compared with the other pathways in both groups. These phenotypes were consistent with the fact that metabolisms of carbohydrates, amino acids, and lipids are the main activities in composting [30].

Pearson correlation analysis revealed that function terms, such as glycolysis/gluconeogenesis, pyruvate metabolism, fatty acid metabolism, synthesis and degradation of ketone bodies, secondary bile acid biosynthesis, and protein export were positively correlated with C/N, OM, and WSS (*p* < 0.01 or *p* < 0.05) but negatively correlated with TKN and pH (Fig. 5b). The abundance of these genes decreased as the composting process progressed. The opposite trend was observed in glycine, serine, and threonine metabolism, streptomycin biosynthesis, and C5-branched dibasic acid metabolism (*p* < 0.01 or *p* < 0.05) (Fig. 5b). Microbes can interact and secrete many enzymes to degrade proteins and various complex carbohydrates in composting, especially during the mesophilic and thermophilic phases, leading to fewer carbohydrates, relatively higher total nitrogen, and higher pH [58].

### Complex microbial agent facilitates *P. ostreatus* colonization on DGW compost

**Fig. 6:**
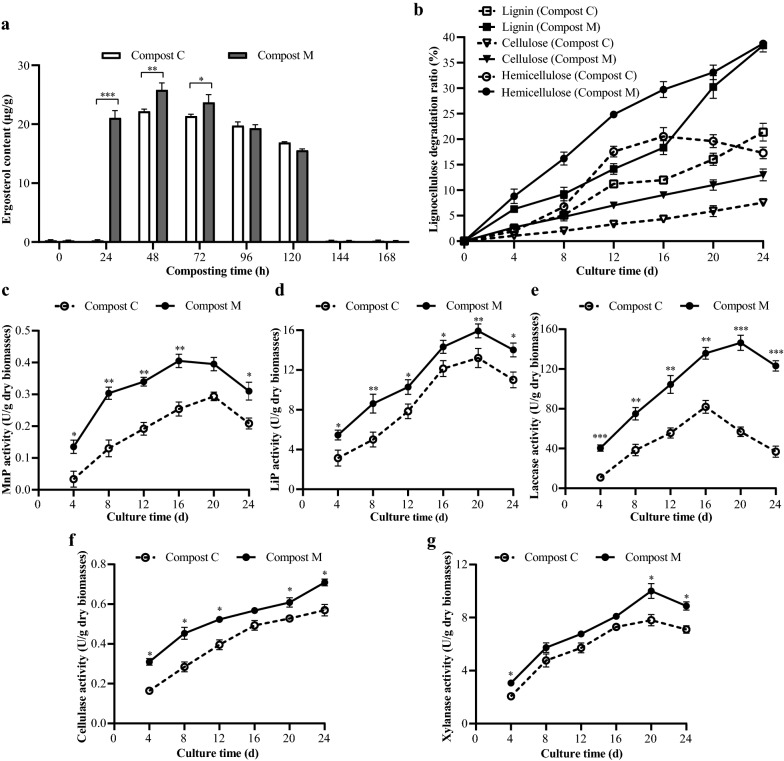
The consortium-based microbial agent improved *P. ostreatus* growth on DGW compost. **a** Ergosterol content of *P. ostreatus* cultured in samples withdrawn at different composting times. **b** Degradation ratios of lignin, cellulose, and hemicellulose in the *P. ostreatus* culture media using the 48 h-composted DGW samples of Compost M and Compost C as substrate. **c**–**g**, Dynamics of MnP activity (**c**), LiP activity (**d**), laccase activity (**e**), cellulase activity (**f**), and xylanase activity (**g**) in the *P. ostreatus* culture media using the 48-h-composted DGW samples of Compost M and Compost C as substrate. Compost M, with microbial inoculation. Compost C, without microbial inoculation. *p < 0.05, **p < 0.01, ***p < 0.001

Although the substrate preparation through composting for champignon cultivation (secondary decomposer) has been developed and improved for more than seventy years, limited information has been gained about substrate preparation through composting for oyster mushroom cultivation (primary decomposer), which possesses entirely different physiological characteristics from champignon [2]. Thus, the effect of the consortium-based microbial agent in composting DGW for *P. ostreatus* cultivation was evaluated using DGW withdrawn at different time intervals as substrates. As shown in Fig. 6a,* P. ostreatus* could not colonization when directly using mixed raw DGW as the substrates because no ergosterol, which is commonly used as a marker to characterize fungal growth [59], was detected in the DGW substrate. By comparison, after 48 h of composting, DGW was the favorable substrate for *P. ostreatus* growth. Its growth slowed down in the substrates as the composting process prolonged, and they stopped growing altogether in the substrate after 144 h of composting (Fig. 6a). Ergosterol content was substantially higher in Compost M than in Compost C, especially in DGW composted for 24 h, indicating that fermentation material cultivation by composting DGW material facilitated the *P. ostreatus* mycelia growth because its growth was faster in the former than in the latter. A possible explanation for this result was the intense microbial activity caused by the inoculant. For example, DGW contains high concentrations of phenolic compounds (3.5–5 mg/g dry matter) and fermented residues that fungi cannot easily use, thus restricting *P. ostreatus* growth in DGW. However, after one day of composting, the total concentration of phenolic compounds decreased to 1.7 mg/g dry matter. As a whole, the inoculation of microbes shortened the DGW composting time and facilitated *P. ostreatus* mycelial growth.

*P. ostreatus* is a famous lignocellulose degradation fungus that employs powerful enzymatic machinery, including LiP, MnP, laccase, cellulose, and xylanase to grow in a wide range of agro-wastes [60]. Thus, the 48 h-composted DGW samples of Compost M and Compost C were used as substrates to cultivate *P. ostreatus*, and the lignocellulose degradation and relative enzymatic activities were measured every 4 d to compare the mycelial growth status. The lignocellulose degradation ratios all increased significantly in Compost M than Compost C (*p* < 0.05 or *p* < 0.01, Fig. 6b). For example, the degradation ratios of lignin, cellulose and hemicellulose were 21.4%, 7.5%, and 17.3% in Compost C after 24 d, while in Compost M they reached 38.4%, 13.0%, and 38.8%, respectively. Correspondingly, higher LiP activity, MnP activity, laccase activity, cellulose activity and xylanase activity were observed throughout the culture period in Compost M (*p* < 0.05, *p* < 0.01 or *p* < 0.001, Fig. 6c–g). These results further suggested that microbes inoculated composting substrate promoted mushroom mycelial colonization.

Five batches of mushroom production experiments were conducted using the 48 h-composted DGW samples of Compost M and Compost C as substrates to cultivate *P. ostreatus*. The final yield of mushroom from Compost M was 0.67 ± 0.05 kg fresh mushroom/kg DGW, a bit higher than the control samples (0.60 ± 0.04 kg fresh mushroom/kg DGW), but without significant difference (*p* > 0.05). However, the harvesting time of the mushroom using substrate from Compost M was 5–7 days earlier than using substrate from Compost C.

## Conclusion

A consortium-based microbial agent comprising five indigenous microorganisms was developed, and its possible use in composting DGW for *P. ostreatus* cultivation was evaluated. Results revealed that inoculation of this microbial agent influenced the physicochemical properties of the DGW compost, the dynamics of the microbial community structures and metabolic functions at different composting phases, and *P. ostreatus* mycelial growth. Thus, the consortium-based microbial agent comprised indigenous microorganisms shows application potential in composting DGW for *P. ostreatus* cultivation and will provide an alternative way for DGW recycling.

## Methods

### Composting materials

Raw DGW was obtained from Golden Seed Winery (Fuyang, Anhui Province, China). Corncob and lime were purchased from the local market. Corncob was crushed to about 0.5 cm in length. The physicochemical properties of raw substrates are listed in Additional file 1: Table S2. *P. ostreatus* was maintained on PDA (potato dextrose agar, filtrate of boiled-potato 200 g L^−1^, glucose 20 g L^−1^, agar, 15 g L^−1^) slants at 4 °C and stored at the School of Life Sciences of Anhui University.

### Screening and identification of microbial inoculum

The compost pile was made of a mixture of 100 kg of DGW and corncob. The 7:3 ratio of DGW and corncob were mixed to adjust the C/N to 30‒35. Lime was added to the composting substrate to adjust the pH to 6.5 [8]. The moisture was adjusted to 65% with tap water. The composting was conducted in a compartment equipped with ventilating machines. Samples from naturally composting of 72 h (in thermophilic phase, > 70 °C) were withdrawn and divided into two parts. One part was sent for 16S rDNA high-throughput sequencing. The other part was used for strain isolation. Briefly, samples were suspended in sterile PBS buffer and diluted via the standard dilution-to-extinction method to 10^–8^ and then spread on Luria–Bertani (LB) agar plates (LB, tryptone 10 g L^−1^, yeast extract 5 g L^−1^, NaCl 10 g L^−1^, Agar, 10 g L^−1^) and incubated at 55 °C for 24 h [14]. Colonies grew on the plates were picked and individually cultured in liquid LB medium at 55 °C on a rotary shaker for 24 h at 180 rpm. Each strain was identified based on the 16S rRNA gene, which was amplified using the primers Bact-27F (5ʹ-AGAGTTTGATCMTGGCTCAG-3ʹ) and Bact-1492R (5ʹ-GGTTACCTTGTTACGACTT-3ʹ). The obtained sequence was blasted in the Eztaxon database (https://www.ezbiocloud.net/) to match the most closely related species [61]. The strains used for developing the consortium-based microbial agent were selected based on their physiological functions, as reported in the references. The phylogenetic tree of the five screened microorganisms was constructed using the MEGA 7 program [62] based on the maximum likelihood method with 1000 bootstrap replicates.

### Composting process and sampling

Five strains employed were individually cultivated in 2.4 L LB medium to the logarithmic growth phase. Cells were mixed and withdrawn by centrifugation, resuspended in 12 L sterile water, and used as inoculants. The composting materials were prepared as described above and segregated into two parts: one part was used for microbial inoculation composting (Compost M), the other was utilized as the control composting (Compost C). Compost M was inoculated with 0.2% (v/m) of the inoculants. The same volume of sterile water was added to Compost C as the control. Each experiment was performed in triplicate, and each repetition contained 200 kg of the composting materials. The mixed materials were stacked to form trapezoidal piles 80 cm width at the top, 150 cm at the bottom, and 60 cm in height. The composting lasted for 186 h in compartments equipped with ventilating machines. Heaps were turned and thoroughly mixed every 24 h. A nine-point sampling method was employed for the sample and data collection to obtain representative results. A total of 1 kg of the sample was collected every 24 h and used to analyze physicochemical properties and utilized as the *P. ostreatus* cultivation substrate.

Samples collected at 18, 72, and 168 h, which belonged to the mesophilic, thermophilic, and cooling phases, were chosen and numbered in Compost M and Compost C for 16S rDNA high-throughput sequencing analysis. M1, M2, and M3 represented the samples from the mesophilic, thermophilic, and cooling phases of Compost M, respectively, whereas C1, C2, and C3 represented the samples from Compost C, respectively.

### Physicochemical analysis

The temperature was measured using an electronic thermometer probe by inserting it into the center of the composting heap with three replicates every 6 h. Other determinations were repeated three times and conducted every 24 h. pH was measured using a pH meter after mixing 10 g of composting samples with 100 mL deionized water [29]. The TKN and organic matter (OM) were determined via the Kjeldahl method (GB/T 6432–2018) and the dry combustion method (NY/T 304–1995), respectively. The WSS was measured via the anthrone sulfuric acid colorimetric method according to Laurentin and Edwards [63].

### *P. ostreatus* cultivation and ergosterol content determination

The water content and pH of composting samples collected at different time points were adjusted to 65% and 7.0, respectively, and then autoclaved at 115 °C for 20 min [64, 65]. Five plugs (5 mm in diameter) of *P. ostreatus* actively growing on the PDA plate at 25 °C were inoculated into 100 mL liquid PDA medium and grown at 25 °C for 4 d with shaking at 120 rpm. The mycelia were then homogenized twice at 3,000 rpm for 5 s and used as the seed to inoculate into the sterile DGW composting material (5%, v/w) and cultured in the dark at 25 °C and 80% humidity.

The *P. ostreatus* mycelial biomass was determined by measuring the content of ergosterol after 10 days of cultivation [66]. Three bags of cultures were withdrawn, dried at 60 ℃ to a constant weight, crushed, and sifted. Ergosterol was extracted by saponification reaction. In brief, samples were mixed with 20% NaOH solution, incubated at 85 °C for 2 h, and centrifuged at 2,000 × *g* for 20 min. Next, the precipitate was added with alcohol, thoroughly mixed, and centrifuged to obtain the supernatant. The supernatant was filtered through 0.22 μm filters, and 200 μL of each sample was used to analyze ergosterol content via a high-performance liquid chromatography equipment equipped with an XDB C18 column (1250 mm × 4.6 mm, 5 µm; Agilent, Palo Alto, USA) and a UV detector (1260 DAD) at 30 °C. The eluting buffer was methanol. The flow rate was 1.0 mL/min.

The wrapped substrate bags (1.5–1.7 kg) were cultured in a humidity-, temperature-, and light-controlled production house to evaluate the final mushroom yield. The mushroom production was closely monitored until the end of the third flush. The mushroom yield was calculated as the wet weight of kilogram fresh mushroom per kilogram substrate.

### Lignocellulose contents and enzymatic activities during *P. ostreatus* colonization

The lignocellulose contents and relative enzymatic activities of DGW compost inoculated with *P. ostreatus* were estimated to evaluate the performance of *P. ostreatus* cultivated in the 48 h DGW composting samples. Three bags were withdrawn everyfour days. Twenty grams of the samples were randomly collected and transferred to a flask containing 100 mL deionized water and blended at 4 °C overnight and away from light, respectively. The mixtures were filtered for removing residues and further separated for the supernatant to measure enzymatic activities by centrifugation at 8000 × *g* for 10 min. Laccase activity of the supernatant was determined with 2,2'-azino-bis(3-ethylbenzothiazoline-6-sulfonate) (ABTS) (0.5 mM) as the substrate according to Bourbonnais and Paice [67]. Lignin peroxidase (LiP) activity was carried out based on the oxidation of veratryl alcohol to veratraldehyde as described by Arora [68]. Manganese peroxidase (MnP) was measured in the presence of MnSO_4_ (0.5 mM), 2,6-dimethylphenol (2,6-DMP) (1.0 mM) and H_2_O_2_ according to Wariishi [69]. Xylanase activity of the supernatant was estimated firstly using xylan solution and then adding 3,5-dinitrosalicylic acid (DNS) as described by Irfan [70]. Cellulase activity was carried out by measuring the reducing sugars by the method described by González Bautista [71].

The cellulose, hemicellulose, and lignin contents were determined on an XD-CXW-10 fiber analyzer according to the method shown by Zang [72]. Among which, the hemicellulose content was calculated by subtraction of the acid-detergent fiber (ADF) from the neutral-detergent fiber (NDF). The cellulose content was estimated by subtraction of the acid-detergent lignin (ADL) from the NDF. The lignin content was calculated as the difference between the ADL and the ash content.

### Genomic DNA extraction and high-throughput sequencing

Total microbial genomic DNA of composted DGW samples was extracted using the DNeasy PowerSoil Kit (Qiagen, Germany) following the manufacturer’s instructions. The quantity and quality of extracted DNA were analyzed using a NanoDrop ND-1000 spectrophotometer (Thermo Fisher Scientific, Waltham, MA, USA) and agarose gel electrophoresis, respectively. PCR amplification of the V3–V4 regions of bacterial 16S rRNA genes was performed using the forward primer 338F (5ʹ-ACTCCTACGGGAGGCAGCAG-3ʹ) and the reverse primer 806R (5ʹ-GGACTACHVGGGTWTCTAAT-3ʹ). Sample-specific 7 bp barcodes were incorporated into the primers for multiplex sequencing. Final PCR amplicons were purified with AMPure beads (Beckman Coulter, Indianapolis, IN) and quantified using the PicoGreen dsDNA assay kit (Invitrogen, Carlsbad, CA, USA). After the individual quantification step, amplicons were pooled in equal amounts, and paired-end 2 × 300 bp sequencing was performed using the Illumina MiSeq platform with MiSeq reagent kit v3 at Shanghai Personal Biotechnology Co., Ltd (Shanghai, China). A negative control to verify possible exogenous contamination was simultaneously set up according to the manufacturer’s instructions.

### Sequence analysis

Quality check of sequencing data was performed using QIIME2 2019.4 [73] with slight modifications according to the official tutorials (http://docs.qiime2.org/2019.4/tutorials/). In brief, raw sequencing data were demultiplexed using the demux plugin followed by primers cutting with the cutadapt plugin [74]. The sequence reads were first filtered using DADA2’s recommended parameters (i.e., an expected error threshold of 2 combined with the trimming of 10 nucleotides from the start and end of each read). Filtered reads were then de-replicated and de-noised using DADA2 default parameters [75]. De-replication combines identical reads into unique sequences and constructs consensus quality profiles for each combined lot of sequences; the consensus quality profiles then inform the de-noising algorithm, which infers error rates from samples and removes identified sequencing errors from the samples [75]. Nonsingleton amplicon sequence variants (ASVs) were aligned using the MAFFT 7.0 software [76] (via q2-alignment) and used to construct a phylogeny with FastTree 2 [77] based on an approximately maximum likelihood method (via q2-phylogeny). Taxonomy was assigned to ASVs by using the classify-sklearn naïve Bayes taxonomy classifier in feature-classifier plugin [78] based on the SILVA Release 132 database (https://www.arb-silva.de/documentation/release-132) [79].

### Bioinformatics analysis

Sequence data analyses were mainly performed using QIIME2 2019.4 and R packages (vision 3.2.0). ASV-level *α*-diversity indices, such as Chao1 richness estimator, Observed species, Shannon diversity index, and Simpson index, were calculated using the ASV table in QIIME2, and visualized as box plots. *β*-Diversity analysis was performed to investigate structural variations in microbial communities across samples by using Jaccard metrics [80], Bray–Curtis metrics [81] and UniFrac distance metrics [82] and visualized via principal coordinate analysis (PCoA) and nonmetric multidimensional scaling (NMDS) [83]. The significance of differentiation of microbiota structure among groups was assessed by ANOSIM (analysis of similarities) using QIIME2 [84]. Taxonomic compositions and abundances were visualized using MEGAN [85] and GraPhlAn [86].

Microbial functions were predicted using PICRUSt2 (Phylogenetic investigation of communities by reconstruction of unobserved states) [87] upon Kyoto Encyclopedia of Genes and Genomes (KEGG) database (https://www.kegg.jp/). Level 3 KEGG ortholog functions about the relative abundance of metabolic functions were drawn in a heatmap using the “pheatmap” package of the R software (version 3.6.3) [88]. RDA was conducted using Canoco (version 5.0.2) to reveal relationships among multiple variations between environmental factors and community compositions and functions [89].

### Statistical analysis

All experimental data are presented as mean ± standard deviation. SPSS analysis (Statistical Product and Service Solutions 24.0 Windows, SPSS Inc, Chicago, USA) was used to investigate the statistical significance of the physicochemical properties of compost and the relative abundance of metabolic functions. The mean values of the samples of different cultivations were compared by ANOVA tests. Duncan’s multiple range test was used for means separation. The level of significance was set at *p* < 0.05. Other statistical significance was evaluated through one-way ANOVA, followed by Student’s *t*-test with GraphPad Prism 7.0. *p* < 0.05 was considered statistically significant.

## Supplementary Information

**Additional file 1:** **Table S1**. The identification results of five microorganisms screened for microbial inoculation from DGW composting. **Table S2**. The physicochemical properties of raw substrates. **Fig. S1**. Effect of microbial inoculation on bacteria community diversity during DGW composting. **Fig. S2** The level 1 KEGG microbial ortholog function profiles in different composting phases during DGW composting.

## Data Availability

The raw metagenomic datasets are publicly available in National Center for Biotechnology Information (NCBI) Sequence Read Archive (BioProject ID: PRJNA723141). The DNA sequence of the Internal Transcribed Spacer (ITS) rRNA gene for molecular identification of *P. ostreatus* is deposited in the GenBank database (the Accession No. OL308083).
