# Supplementary material for: Development of a consortium-based microbial agent beneficial to composting of distilled grain waste for Pleurotus ostreatus cultivation
Source: Biotechnol Biofuels. 2021 Dec 17;14:242. doi: 10.1186/s13068-021-02089-4 (PMC8684267; doi:10.1186/s13068-021-02089-4)
Supplement: Supplementary file 1 — Additional file 1: Table S1. The identification results of five microorganisms screened for microbial inoculation from DGW composting. Table S2. The physicochemical properties of raw substrates. Fig. S1. Effect of microbial inoculation on bacteria community diversity during DGW composting. Fig. S2 The level 1 KEGG microbial ortholog function profiles in different composting phases during DGW composting. [file 13068_2021_2089_MOESM1_ESM.docx]

**Additional file 1**

**Development of a compound microbial agent beneficial to composting** **of distilled grain waste for *Pleurotus ostreatus* cultivation**

Sibao Wu^a,b,#^, Rongrong Zhou^a,b,#^, Yuting Ma^a,b^, Yong Fang^a,b^, Guopai Xie^c^, Xuezhi Gao^d^, Yazhong Xiao^a,b^, Juanjuan Liu^a,b,*^, Zemin Fang^a,b,*^

a, School of Life Sciences, Anhui University, 230601 Hefei, Anhui, China;

b, Anhui Key Laboratory of Modern Biomanufacturing, 230601 Hefei, Anhui, China;

c, Anhui Golden Seed Winery Co., LTD, 341200 Fuyang, Anhui, China

d, Livestock and poultry breeding service center of Fuyang city, 341200 Fuyang, Anhui, China

# These two authors contributed equally to this work

* Corresponding authors

Phone/Fax: +86-551-63861063

E-mail: liu_juan825@ahu.edu.cn; zemin_fang@ahu.edu.cn

Table S1 The identification results of five microorganisms screened for microbial inoculation from DGW composting.

| Number | Hit taxon name | Hit strain name | Accession | Identity  (%) | Similarity (%) | Coverage  (%) | Completeness (%) |
| --- | --- | --- | --- | --- | --- | --- | --- |
| X1 | *Sphingobacterium thermophilum* | CKTN2(T) | AB563783 | 98.85 | 99.13 | 100 | 100.0 |
|  | *Sphingobacterium cibi* | CC-YY411(T) | JN941762 | 94.41 | 93.89 | 96 | 100.0 |
| X2 | *Ureibacillus thermosphaericus* | DSM 10633(T) | AB101594 | 100.00 | 100.00 | 100 | 100.0 |
|  | *Ureibacillus composti* | HC145(T) | DQ348071 | 97.72 | 97.71 | 100 | 100.0 |
|  | *Ureibacillus thermophilus* | HC148(T) | DQ348072 | 97.44 | 97.14 | 99 | 100.0 |
| X3 | *Pseudoxanthomonas* AZNZ_s | J31 | AZNZ01000026 | 100.00 | 100.00 | 100 | 100.0 |
|  | *Pseudoxanthomonas suwonensis* | 4M1(T) | AY927994 | 99.06 | 98.56 | 98 | 100.0 |
| X4 | *Geobacillus thermodenitrificans subsp. calidus* | F84b(T) | EU477773 | 99.64 | 99.64 | 89 | 94.7 |
|  | *Geobacillus thermodenitrificans subsp. thermodenitrificans* | KCTC3902(T) | CP017694 | 99.46 | 99.46 | 94 | 100.0 |
|  | *Geobacillus subterraneus subsp. subterraneus* | KCTC3922(T) | CP014342 | 98.92 | 98.99 | 94 | 100.0 |
| X5 | *Aeribacillus pallidus* | KCTC3564(T) | CP017703 | 99.93 | 99.93 | 94 | 100.0 |
|  | *Aeribacillus composti* | N.8(T) | LT594972 | 99.72 | 99.59 | 92 | 98.1 |

Table S2 The physicochemical properties of raw substrates.

| Materials | Moisture content (%) | total nitrogen (%) | total organic carbon (%) | C/N | pH |
| --- | --- | --- | --- | --- | --- |
| Distiller’s grain waste | 60.32±2.63 | 2.96±0.21 | 52.62±1.33 | 18.56±1.12 | 3.51±0.22 |
| Corn cob | - | 0.52±0.03 | 53.27±2.01 | 99.55±1.18 | - |
| Distiller’s grain Waste + 30% corn cob | 65.51±1.21 | 1.43±0.08 | 51.99±1.56 | 34.52±0.87 | 6.55±0.26 |


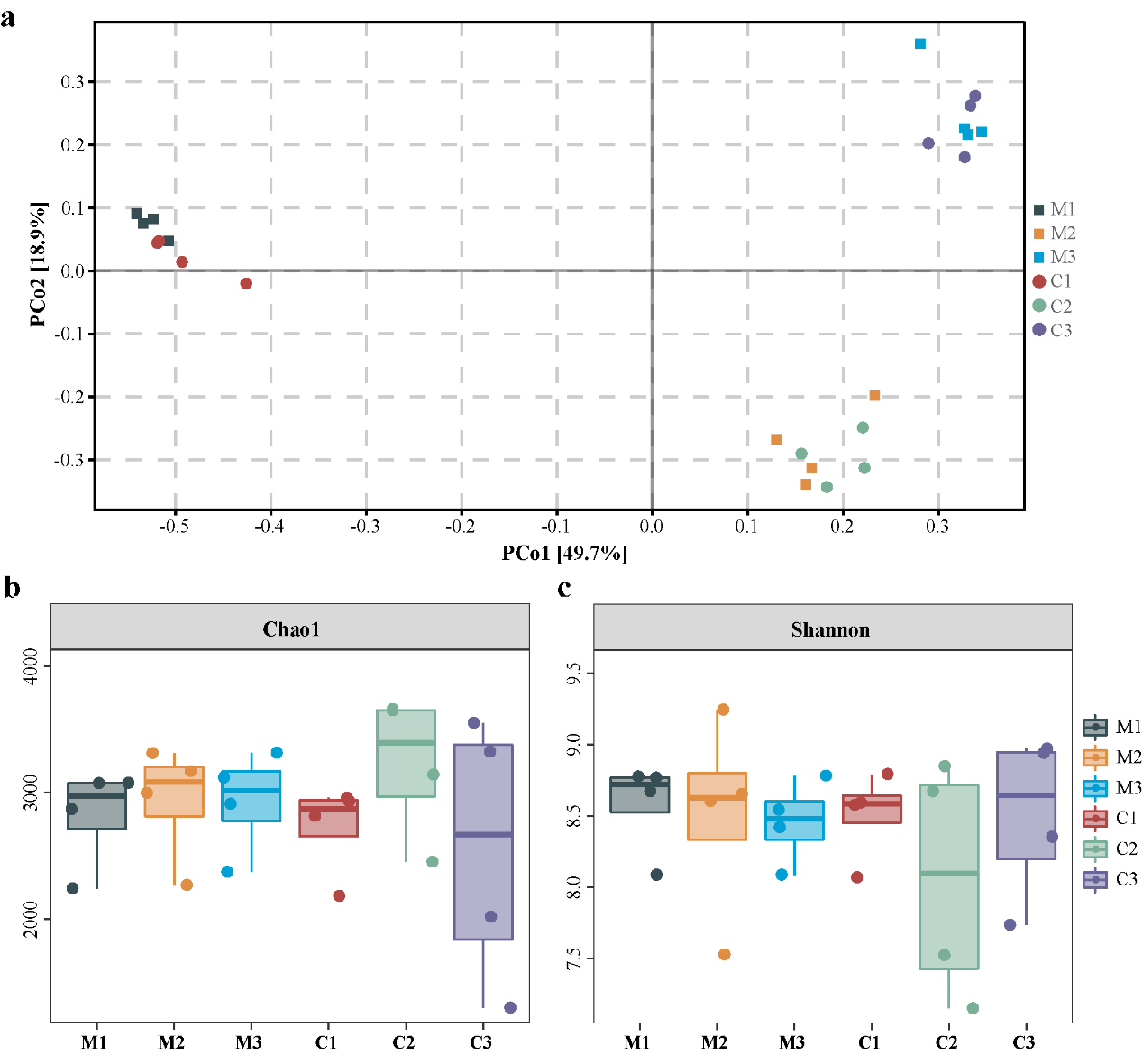


**Fig. S1** **Effect of microbial inoculation on bacteria community diversity during DGW composting.** (a), Principle coordinates (PCoA) analysis. (b), Chao 1 index. (c), Shannon index. M1, M2, and M3 represent the samples from mesophilic, thermophilic, and cooling phases of Compost M, respectively, while C1, C2, and C3 represent the samples from mesophilic, thermophilic, and cooling phases of Compost C.


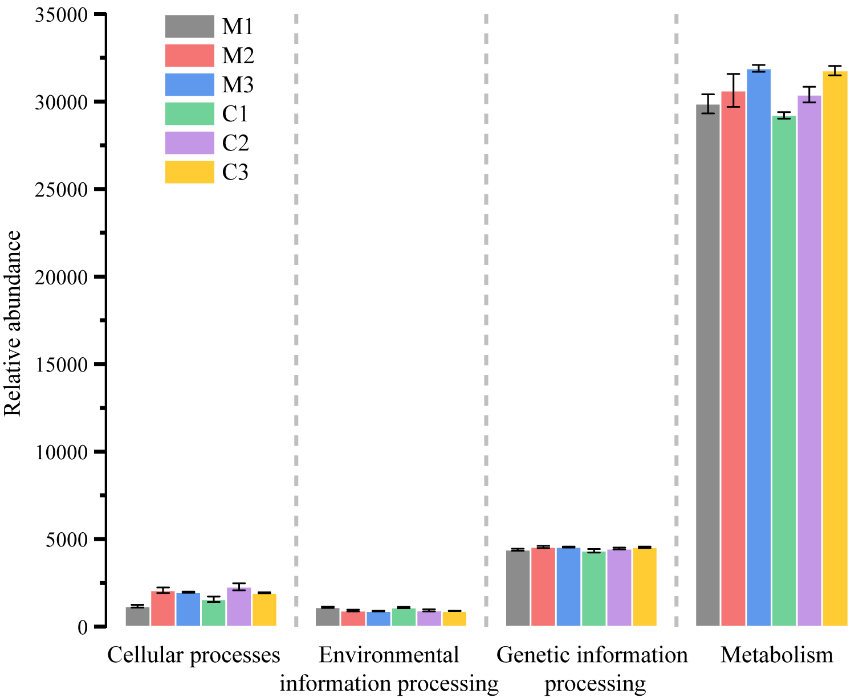


**Fig. S2** The level 1 KEGG microbial ortholog function profiles in different composting phases during DGW composting. M1, M2, and M3 represent the samples from mesophilic, thermophilic, and cooling phases of Compost M, respectively, while C1, C2, and C3 represent the samples from mesophilic, thermophilic, and cooling phases of Compost C.
